# Supplementary material for: Stress during pubertal development affects female sociosexual behavior in mice
Source: Nat Commun. 2024 Apr 30;15:3610. doi: 10.1038/s41467-024-47300-w (PMC11061123; doi:10.1038/s41467-024-47300-w)
Supplement: Supplementary file 1 — Supplementary Information [file 41467_2024_47300_MOESM1_ESM.pdf]

Supplemental information

Stress during pubertal development affects female sociosexual behavior in mice

Supplementary figures

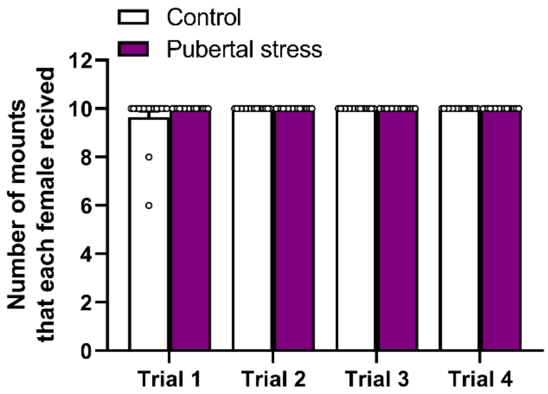

Fig s1. Number of mounts that each female received by the stimulus males during the testing for lordosis behavior. RM two ANOVA; Group effect: F(1,31)=1.78, p=0.19 ; trial effect: F(3,93)=1.78, p=0.156: interaction: F(3,93)=1.78, p=0.156. Control: n=17; Pubertal stress n=16. Data is presented as mean ± SEM. Source data are provided as a Source Data file.

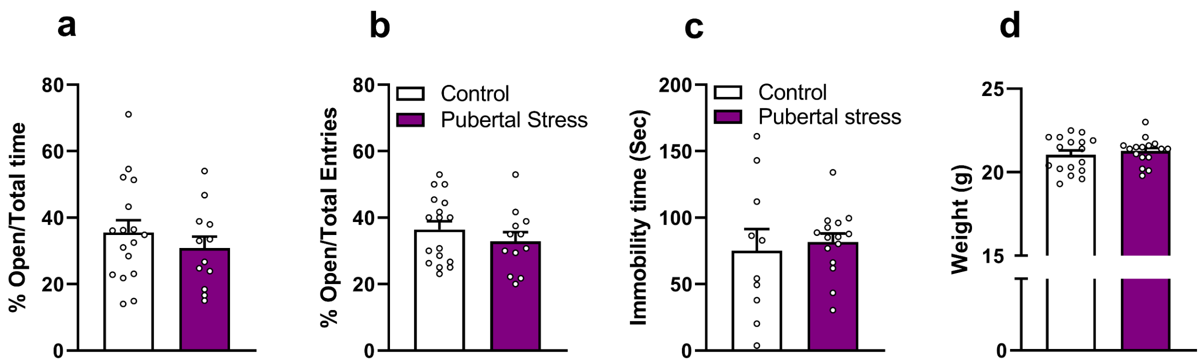

Fig s2. Exposure to pubertal stress in female mice had no effect on state of anxiety, depression-like behaviors, or body weight. (a) Percentage of time spent in the open arms of the elevated plus maze in the control group (n=17) and females exposed to pubertal stress (n=12). Unpaired t test (two-tailed): t(27)=0.90, p=0.375 **(b)** Percentage of entries into the open arms. Two-tailed unpaired t test: t(27)=0.956. n=17 for the control and n=12 for pubertal stress group. **(c)** Time spent immobile in the forced swim test for the control subjects (n=10) and females subjected to pubertal stress (n=15). Unpaired t test (two-tailed) t(23)=0.431, p=0.67. **(d)** Weight of the females at the age of two months. Two-tailed unpaired t test: t(31)=0.66, p=0.512. n=17 and 16 for the control and pubertal stress groups, respectively. All data are presented as mean ± SEM. Source data are provided as a Source Data file.

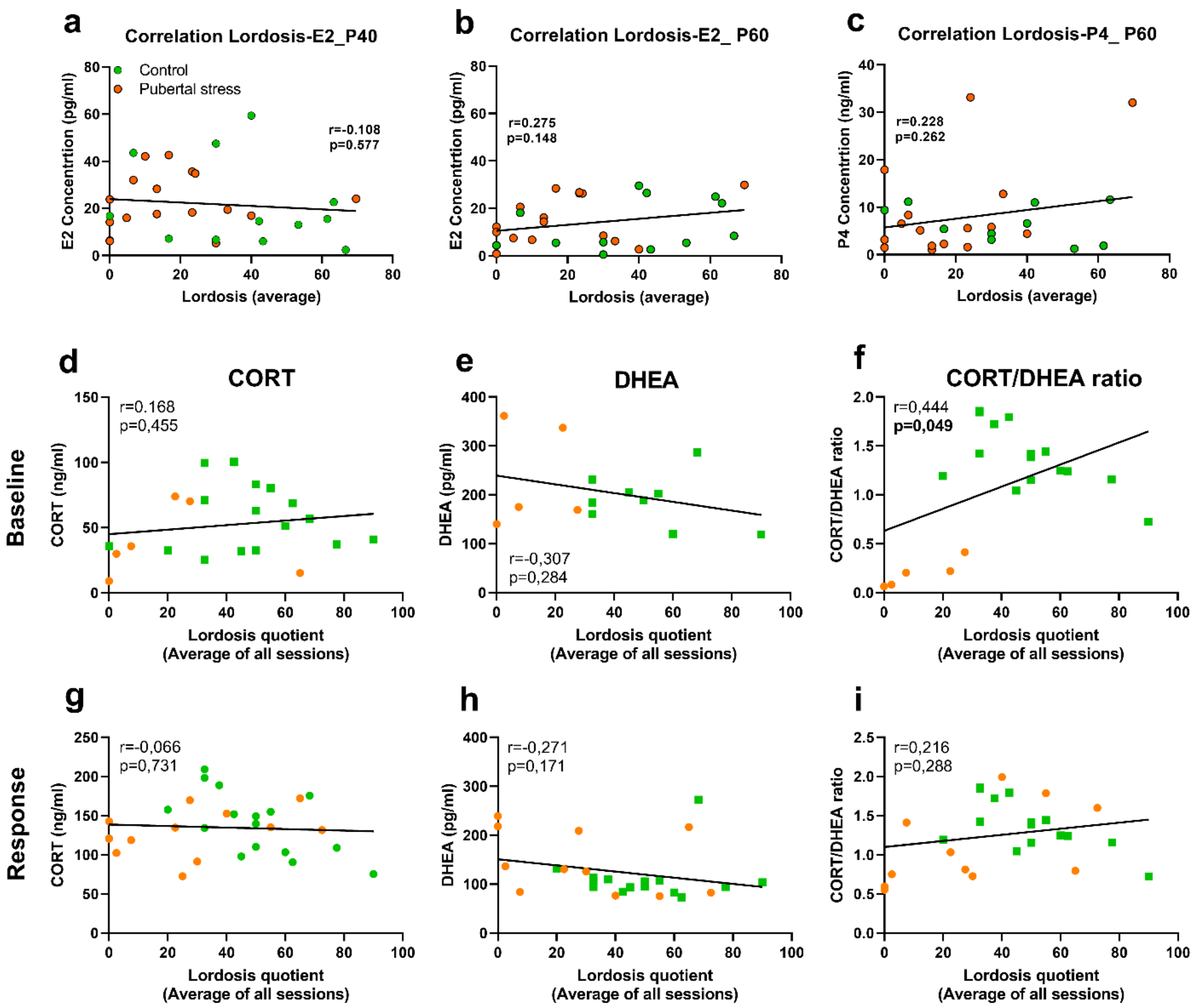

Fig s3: Correlations between sexual receptivity and steroid hormones in control and pubertally stressed female mice. Correlation between lordosis and estradiol concentrations at the age of P40 **(a)** and P60 **(b)**. **(c)** Correlation between progesterone and lordosis at the age of P60. Correlation between lordosis and baseline corticosterone **(d)**, DHEA **(e)**, and Corticosterone/DHEA ratio. Correlation between lordosis and corticosterone(**g**), DHEA **(h)** and corticosterone/DHEA ratio **(i)** following activation of the HPA axis by exposure to the elevated plus maze. All tests are two-tailed. Data are presented as mean ± SEM. Source data are provided as a Source Data file.

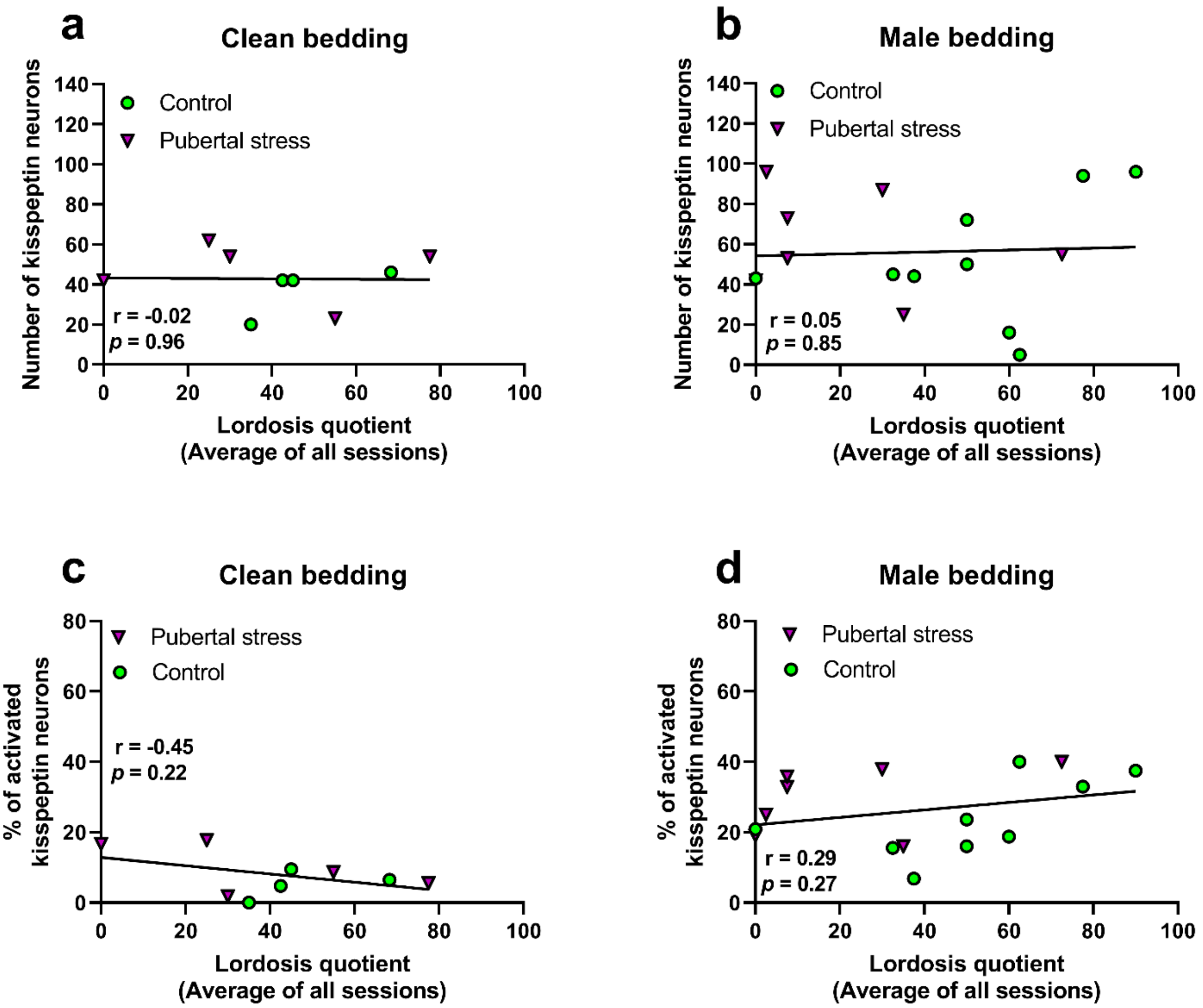

Fig s4. Number of RP3V kisspeptin neurons or their activation, measured by Fos expression, do not correlate with lordosis behavior. Correlation between lordosis quotient and the number of kisspeptin neurons in females exposed to clean **(a)** or male bedding **(b)**. Correlation between lordosis quotient and the percentage of activated kisspeptin neurons following exposure to clean **(c)** or male bedding **(d)**. All tests are two-tailed. Data are presented as mean ± SEM. Source data are provided as a Source Data file.

	Control		Pubertal stress		F(df1,df2); p Value		
	Clean bedding	Male bedding	Clean bedding	Male bedding	Group effect	Bedding effect	Interaction
**ACo**	143.0±11.5 (n=4)	201.1±19.8 (n=9)	155±27.9 (n=5)	186.0±21.9 (n=4)	F(1,18)=0.003; p=0.95	F(1,18)=3.4; p=0.08	F(1,18)=0.32; p=0.57
**BLA**	92.5±13.7 (n=4)	106.5±13.9 (n=9)	94.6±14.7 (n=5)	122.3±12.3 (n=7)	F(1,21)=0.3; p=0.56	F(1,21)=1.9; p=0.18	F(1,21)=0.2; p=0.65
**MeA**	124.0±13.7 (n=4)	201.2±14.3 (c=9)	191.5±35.7 (n=4)	191.0±12.5 (n=6)	F(1,19)=2.17; p=0.16	F(1,19)=3.9; p=0.06	F(1,19)=4.0; p=0.06
**MePD**	164.5±8.7 (n=4)	210.7±18.1 (n=9)	160.6±21.8 (n=5)	224.7±23.9 (n=7)	F(1,21)=0.05; p=0.82	F(1,21)=6.2; **p=0.02**	F(1,21)=0.16; p=0.69
**MePV**	195.0±20.4 (n=4)	240.8±24.4 (n=9)	194.0±37.5 (n=5)	245.7±29.5 (n=7)	F(1,21)=0.004; p=0.94	F(1,21)=2.5; p=0.12	F(1,21)=0.009 ; p= 0.92
**BSTpm**	71.7±7.9 (n=4)	199.9±19.8[a] (n=8)	131.4±11.6 (n=5)	164.2±12.5[b] (n=5)	F(1,18)=0.46; p=0.50	F(1,18)=21.1; **p=0.0002**	F(1,18)=7.41; **p=0.014**
**mPOA**	65.2±16.4 (n=4)	159.4±25.3 (n=9)	145.8±17.3 (n=5)	205.7±24.4 (n=6)	F(1,20)=6.03; **p=0.02**	F(1,20)=8.91; **p=0.007**	F(1,20)=0.44; p=0.51
**VMHvl**	18.00±7.82 (n=4)	59.37±8.22[a] (n=8)	26.00±6.05 (n=5)	32.00±1.78[c] (n=6)	F(1,19)=1.798; p=0.196	F(1,19)=10.75; **p=0.004**	F(1,19)=5.99; **p=0.024**

Table s1. Number of Fos immunoreactive neurons in various hypothalamic and limbic nuclei important in female sexual behavior. Control and pubertally stressed female mice displayed similar numbers of Fos immunoreactive neurons following exposure to male bedding in the anterior cortical amygdaloid nucleus (ACo), basolateral amygdala (BLA), postero-dorsal part of the medial amygdala (MePD), postero-ventral part of the medial amygdala (MePV), bed nucleus of the stria terminalis, posteromedial part (BSTpm) and medial preoptic area (mPOA). Statistical difference between the two groups following exposure to male bedding was observed for ventrolateral part of the ventromedial hypothalamus (VMHvl). F and P values indicated in the table were obtained following statistical analyses with two-way ANOVA. a: p< 0.01 vs control subjects exposed to clean bedding (post hoc analysis). b: p< 0.05 vs control subjects exposed to clean bedding (post hoc analysis). c: p< 0.05 vs control subjects exposed to male bedding (post hoc analysis). All values are shown as mean ± SEM. The number of animals used for counting are indicated in the table for each brain nucleus, group and treatment.

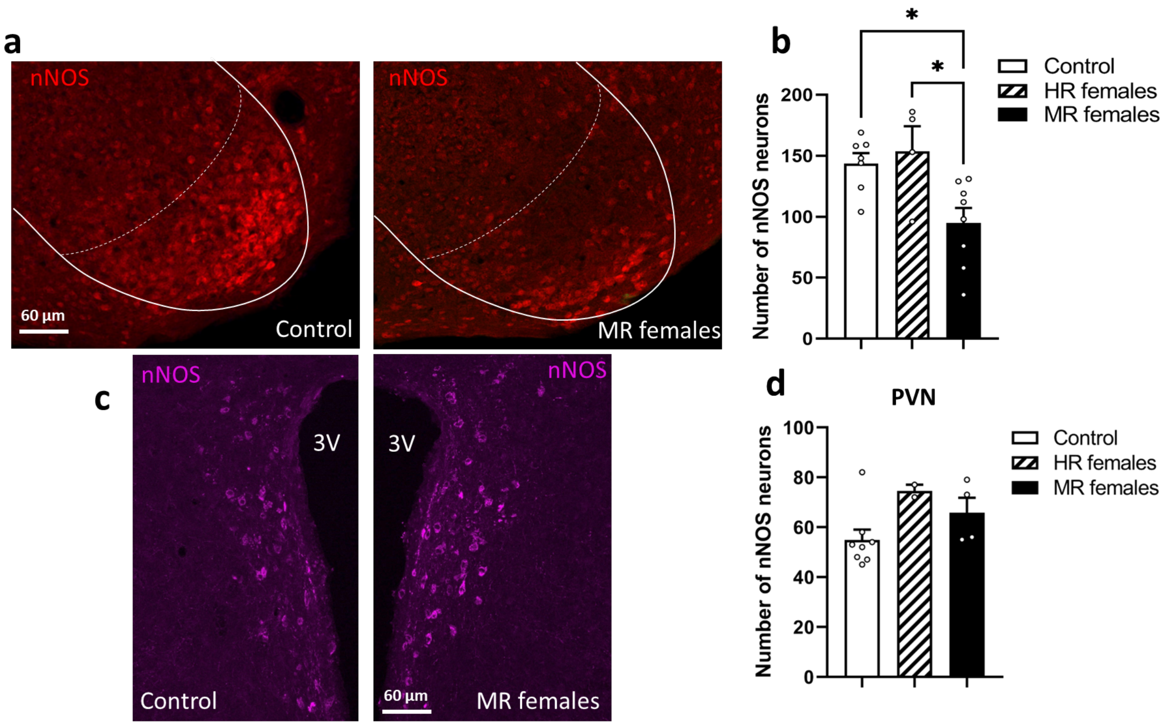

Fig s5. Exposure to stress during puberty reduced the number of VMHvl nNOS neurons without affecting the PVN nNOS population. (a) Representative images of nNOS neurons in the VMHvl in control and MR females that were exposed to pubertal stress. **(b)** Number of nNOS neurons in the control and the two subgroups of females exposed to pubertal stress; minimally receptive (MR) and highly receptive (HR) females. One-way ANOVA; F(2,16)=6.249, p=0.0099. *p=0.0298 (control vs MR females), *p=0.026 (HR vs MR females); Bonferroni's multiple comparisons test. Control (n=7), HR females (n=4), MR females (n=8). **(c)** Representative images of nNOS expression in PVN nucleus in control and MR females. **(d)** Number of nNOS neurons in the PVN in control group (n=8), HR (n=2) and MR (n=4) females. Kruskal-Wallis test; H(3)= 4.386, p=0.106. All values are shown as mean ± SEM. Source data are provided as a Source Data file.

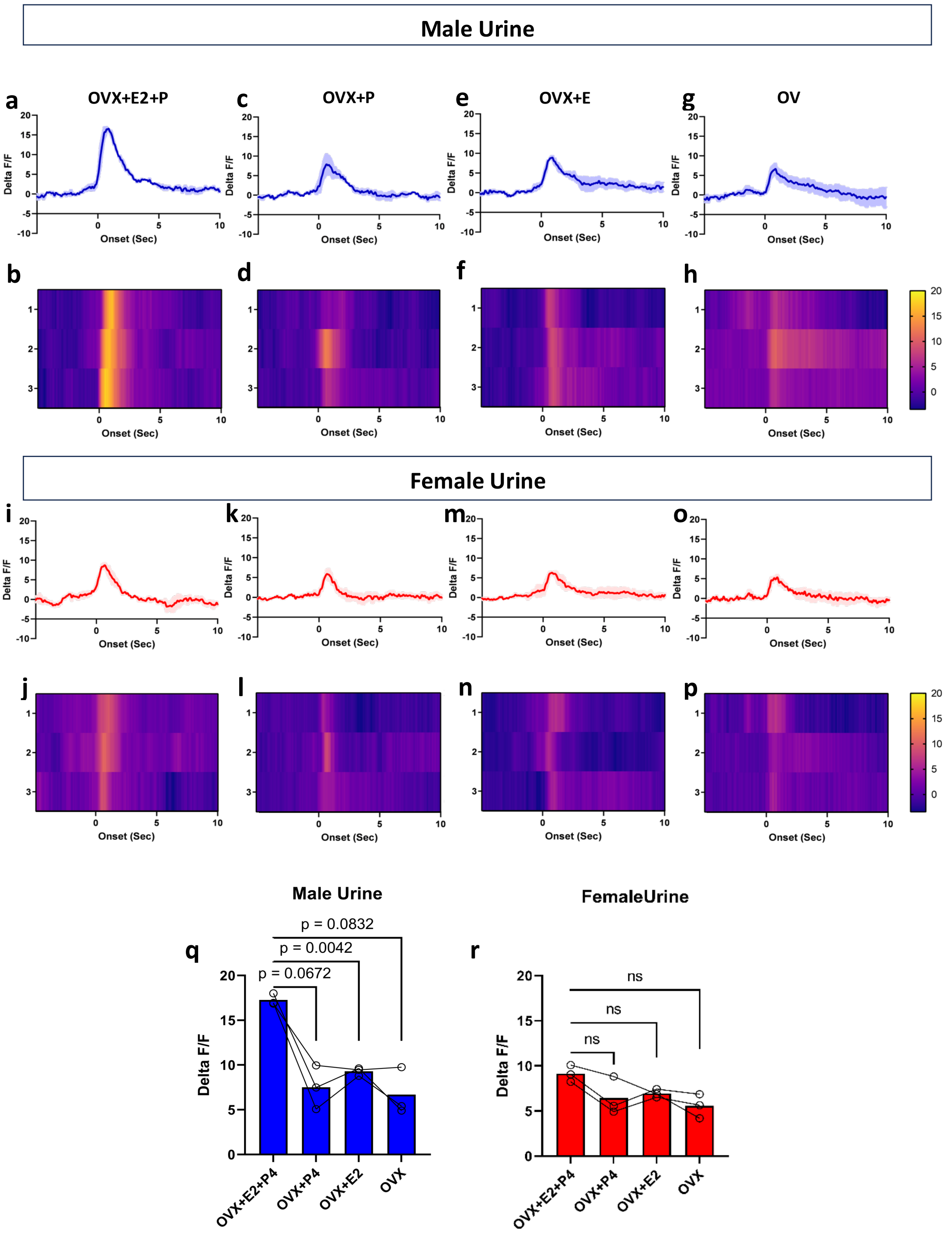

Fig s6. VMHvl nNOS neurons are activated by male olfactory cues and modulated by the synergic effect of estradiol and progesterone. (a, b, i, j) Peri-event time plots and heat maps of nNOS neurons to male urine or female in ovariectomized females that were primed with estradiol and progesterone (OVX+E2+P4). Peri-event time histograms and heat maps of nNOS activation following exposure to male or female urine in ovariectomized females primed with either progesterone (OVX+P4) **(c, d, k, l)**, or estradiol alone (OVX+E2) **(e, f, m, n). (g, h, o, p)** Peri-event time histograms and heat maps of nNOS activation in response to male or female urine following removal of the estradiol implant and a washout period of two weeks. **(q)** Comparison of nNOS activation in response to male urine. RM one-way ANOVA followed by Bonferroni multiple comparison test; $F(1.313, 2.626)=27.83$, $p=0.0178$. **(r)** Comparison of nNOS activation to female urine in different hormonal conditions. RM one-way ANOVA; $F(3,6)=3.636$, $p=0.0837$. All subjects used in this experiment were sexually naive. ns: not significant. OVX: ovariectomized; E2: estradiol; P4: progesterone. Data are presented as mean ± SEM. Source data are provided as a Source Data file.
