# Supplementary material for: Stress during pubertal development affects female sociosexual behavior in mice
Source: Nat Commun. 2024 Apr 30;15:3610. doi: 10.1038/s41467-024-47300-w (PMC11061123; doi:10.1038/s41467-024-47300-w)
Supplement: Supplementary file 3 — Description of Additional Supplementary Information [file 41467_2024_47300_MOESM3_ESM.docx]

**Description of Additional Supplementary Files**

File Name: Supplementary Video 1

Description: Video illustrating the activation of the nNOS neurons located

in the VMHvl in response to different olfactory cues.
