# Supplementary material for: Stress during pubertal development affects female sociosexual behavior in mice
Source: Nat Commun. 2024 Apr 30;15:3610. doi: 10.1038/s41467-024-47300-w (PMC11061123; doi:10.1038/s41467-024-47300-w)
Supplement: Supplementary file 5 — Reporting Summary [file 41467_2024_47300_MOESM5_ESM.pdf]

nature portfolio

Corresponding author(s): Yassine Bentefour
Last updated by author(s): Feb 11, 2024

Reporting Summary

Nature Portfolio wishes to improve the reproducibility of the work that we publish. This form provides structure for consistency and transparency in reporting. For further information on Nature Portfolio policies, see our Editorial Policies and the Editorial Policy Checklist.

Statistics

For all statistical analyses, confirm that the following items are present in the figure legend, table legend, main text, or Methods section.

n/a	Confirmed	
☐	☒	The exact sample size (*n*) for each experimental group/condition, given as a discrete number and unit of measurement
☐	☒	A statement on whether measurements were taken from distinct samples or whether the same sample was measured repeatedly
☐	☒	The statistical test(s) used AND whether they are one- or two-sided<br>*Only common tests should be described solely by name; describe more complex techniques in the Methods section.*
☐	☒	A description of all covariates tested
☐	☒	A description of any assumptions or corrections, such as tests of normality and adjustment for multiple comparisons
☐	☒	A full description of the statistical parameters including central tendency (e.g. means) or other basic estimates (e.g. regression coefficient) AND variation (e.g. standard deviation) or associated estimates of uncertainty (e.g. confidence intervals)
☐	☒	For null hypothesis testing, the test statistic (e.g. *F*, *t*, *r*) with confidence intervals, effect sizes, degrees of freedom and *P* value noted<br>*Give P values as exact values whenever suitable.*
☒	☐	For Bayesian analysis, information on the choice of priors and Markov chain Monte Carlo settings
☒	☐	For hierarchical and complex designs, identification of the appropriate level for tests and full reporting of outcomes
☒	☐	Estimates of effect sizes (e.g. Cohen's *d*, Pearson's *r*), indicating how they were calculated

Our web collection on statistics for biologists contains articles on many of the points above.

Software and code

Policy information about availability of computer code

Data collection	All softwares used are mentioned in the methods section. Synapse essential (fibre photometry recording; TDT); Zen (confocal images acquisition; Zeiss); MultisKan (Absorbance reader for Elisa)
Data analysis	All used software for data analyses are mentioned in the methods section. FIJI (iamge analysis; NIH); Prism 9 (statistial analyses; Graphpad); GuPPy (python toolbox for fibre photometry analysis; https://doi.org/10.1038/s41598-021-03626-9)

For manuscripts utilizing custom algorithms or software that are central to the research but not yet described in published literature, software must be made available to editors and reviewers. We strongly encourage code deposition in a community repository (e.g. GitHub). See the Nature Portfolio guidelines for submitting code & software for further information.

Data

Policy information about availability of data

All manuscripts must include a data availability statement. This statement should provide the following information, where applicable:

- Accession codes, unique identifiers, or web links for publicly available datasets
- A description of any restrictions on data availability
- For clinical datasets or third party data, please ensure that the statement adheres to our policy

The data generated in this study are provided in the supplementary source data file.

Research involving human participants, their data, or biological material

Policy information about studies with human participants or human data. See also policy information about sex, gender (identity/presentation), and sexual orientation and race, ethnicity and racism.

Reporting on sex and gender	Not applicable in this study
Reporting on race, ethnicity, or other socially relevant groupings	Not applicable in this study
Population characteristics	Not applicable in this study
Recruitment	Not applicable in this study
Ethics oversight	Not applicable in this study

Note that full information on the approval of the study protocol must also be provided in the manuscript.

Field-specific reporting

Please select the one below that is the best fit for your research. If you are not sure, read the appropriate sections before making your selection.

☒ Life sciences ☐ Behavioural & social sciences ☐ Ecological, evolutionary & environmental sciences

For a reference copy of the document with all sections, see nature.com/documents/nr-reporting-summary-flat.pdf

Life sciences study design

All studies must disclose on these points even when the disclosure is negative.

Sample size	Sample sizes were not predetermined by statistical methods, but by conventional requirements in the respective fields, and based on our previous experience with studies addressing reproduction in mice (Hellier et al. 2018, Nature communications; Bentefour et al., 2021, Neuroparmacology).<br>The sample size employed a minimum of 3 biological replicates. All sample sizes are listed in each figure legend.
Data exclusions	Mice were excluded from analyses only if the guide cannula, virus expression an/or optic fiber placement were not located in the targeted brain region. This was done to avoid including calcium imaging data from subjects in which we did not record the VMHvl nNOS population. Similarly, anatomical outliers in stereotaxic cannula implantation were not included because they did not receive the pharmacological treatment in the targeted location.
Replication	Most of the experiments, including lordosis, fiber photometry, immunofluorescence for nNOS were successfully replicated in at least 2 separate experiments. All other experiments were performed only once with homogeneous effects.
Randomization	On postnatal day 21, females were randomly assigned to control or pubertal stress group. During behavioral testing, mice were randomly assigned to clean or male bedding to induce c-Fos expression. All animals used in one group were from different litters in order to increase diversity.
Blinding	Blinded data collection was done when possible. However, as Y.B conducted all experiments on his own, data collection for behavioral and pharmacological experiments was not possible. However, all data was analyzed blindly with respect to groups and treatments to avoid any potential bias.

Reporting for specific materials, systems and methods

We require information from authors about some types of materials, experimental systems and methods used in many studies. Here, indicate whether each material, system or method listed is relevant to your study. If you are not sure if a list item applies to your research, read the appropriate section before selecting a response.

Materials & experimental systems

n/a	Involved in the study
☐	☒ Antibodies
☒	☐ Eukaryotic cell lines
☒	☐ Palaeontology and archaeology
☐	☒ Animals and other organisms
☒	☐ Clinical data
☒	☐ Dual use research of concern
☒	☐ Plants

Methods

n/a	Involved in the study
☒	☐ ChIP-seq
☒	☐ Flow cytometry
☒	☐ MRI-based neuroimaging

Antibodies

Antibodies used

-Anti c-Fos (Guinea pig polyclonal) Synaptic systems Cat# 226005; dilution 1/1000 immunohistochemistry and 1/500 immunofluorescence.
-Anti-Kisspeptin 10 (rabbit polyclonal) (Alain Caraty, INRA France); dilution 1/10000 immunohistochemistry.
-Anti-nNOS (Rabbit polyclonal) ThermoFisher Cat# 61-700; Dilution 1/500 immunofluorescence.
-FluoTag-X2 anti-GFP labeled with Atto488 (NanoTag Biotechnologies GmBH Cat# N0304-At488); Dilution 1/500 immunofluorescence.
-Anti-GR (Rabbit polyclonal), Proteintech Cat# 24050-1-AP; dilution 1/200 immunofluorescence.
-Anti-ER alpha (rabbit polyclonal) Millipore Cat#06-935; dilution 1/2000 immunofluorescence
-Anti-PR (rabbit polyclonal) Dako Cat#A0098 ; dilution 1/100 immunofluorescence
-Anti-pnNOS (Rabbit polyclonal) ThermoFisher Cat# PA1032; dilution 1/500 immunofluorescence.
- Anti-guinea pig (goat) biotinylated antibody; Vector Labs Cat# BA-7000; dilution 1/1000 immunofluorescence.
-Anti-Rabbit (goat) biotinylated antibody; Jackson immunoResearch Cat# 111-065-003; dilution 1/1000 immunohistochemistry.
-Anti-rabbit (goat) coupled with Alexa Fluor-546; ThermoFisher Cat# A-11010; dilution 1/500.
-Anti-guinea pig (goat) coupled with Alexa Fluor-488; ThermoFisher Cat# A-11073; dilution 1/500.
-Anti Rabbit (goat) coupled with Alexa Fluor-594; Jackson immunoResearch Cat# 111-585-003; dilution 1/500.
- Unconjugated FAB anti rabbit (goat), Jackson ImmunoResearch Cat# 111-007-003; dilution 30 ug/ml.

Validation

Information on validation of the anti-kisspeptin antibody is reported previously (by Franceschini et al. 2006: DOI: 10.1016/j.neulet.2006.03.039).

All other primary antibodies in this study are commercially available and validated by the manufacturers or used in previous studies.
-Anti-nNOS (ThermoFisher Cat# 61-700) has been published (10.3389/fncir.2012.00065), (DOI: 10.1038/s41467-017-02797-2) and validated for use in immunofluorescence (www.thermofisher.com/antibody/product/nNOS-Antibody-Polyclonal/61-7000).

-Anti-Fos (Synaptic systems Cat# 226005) antibody has been published (DOI: 10.1016/j.neuron.2020.01.037) and validated for use in immunofluoresence and immunohistochimistry (https://sysy.com/product/226005).

-Fluotag-X2 anti-GFP-Att488 (NanoTag Biotechnologies GmBH, Cat# N0304-At488) is validated that it recognizes GFPs (green fluorescent protein) like EGFP, mEGFPa and all others deriviaties. The manufacturer mentions that this antibody is suitable for immunofluoresence applications (https://nano-tag.com/product/fluotag-x2-anti-gfap/). (https://doi.org/10.3390/ijms24087294).

-Anti GR (Proteintech Cat# 24050-1-AP) antibody is validated by the vendor to use in westernbloat, and immunohistochimistry (https://www.ptglab.com/products/NR3C1-Antibody-24050-1-AP.htm). it has been used in multiple publications like (https://doi.org/10.1073/pnas.1411356112).

-Anti ER alpha (Millipore Cat#06-935) polyclonal antibody detects estrogen receptor alpha, and has been tested for use in immunocytochemistry, immunohistochimistry, immunoprecipitation and western blotting (https://www.merckmillipore.com/INTL/en/product/Anti-Estrogen-Receptor-Antibody,MM_NF-06-935#). It has been used in many studies, like (DOI: 10.1172/JCI74726) and (DOI: 10.1371/journal.pone.0090451).

-Anti-PR (Dako Cat#A0098) antibody has been validated by the vendor for immunohistochimistry applications (https://www.labome.com/product/Dako/A0098.html) as well as in a seprate immunohistochimistry knockout validation (DOI: 10.1016/j.celrep.2020.03.060)
-Anti-pnNOS (ThermoFisher Cat# PA1032) antibody was verified by cell treatment to ensure that the antibody binds to the antigen stated, as mentioned by the vendor (https://www.thermofisher.com/antibody/product/Phospho-nNOS-Ser1417-Antibody-Polyclonal/PA1-032). It was used in multiple studies (DOI: 10.1210/en.2010-0007) (DOI: 10.1016/j.freeradbiomed.2022.11.040).

Animals and other research organisms

Policy information about studies involving animals; ARRIVE guidelines recommended for reporting animal research, and Sex and Gender in Research

Laboratory animals

As described in the methods juvenile females (P21) were used to induce pubertal stress. Behavioral experiments were performed on them when they reached adult age (P60). Wild-type C57BL/6J and nNOS::Cre (C57Bl/6J background; JAX stock #017526) were used in

our experiments.
Animals were kept under standard laboratory conditions until crossed to generate the experimental subjects. Experimental and stimulus animals were housed under conditions of controlled temperature (22 ± 2 °C) and lighting (12-h light, 12-h dark cycle; lights off at 08:00h and lights on at 20:00h) with food and water available ad libitum. Stimulus males were housed individually, whereas stimulus females were kept group housed in 3 to 4 subjects per cage. All behavioral tests were conducted in the dark phase of the light cycle.

Wild animals

This study did not involve wild animals.

Reporting on sex

Only female mice were used for this study as experimental subjects. Males were used as stimuli or for collecting urine that was used for olfactory stimulation.

Field-collected samples

This study did not involve samples collected from the field.

Ethics oversight

All procedures were conducted following the guidelines for the care and use of laboratory animals and were approved by the ethics committee of the University of Liège.

Note that full information on the approval of the study protocol must also be provided in the manuscript.

Plants

Seed stocks

Not applicable in this study

Novel plant genotypes

Not applicable in this study

Authentication

Not applicable in this study
